# Extended stable equilibrium invaded by an unstable state

**DOI:** 10.1038/s41598-019-51064-5

**Published:** 2019-10-22

**Authors:** Camila Castillo-Pinto, Marcel G. Clerc, Gregorio González-Cortés

**Affiliations:** Physics Department and Millennium Institute for Research in Optics, Facultad de Ciencias Físicas y Matemáticas, Universidad de Chile, Casilla 487-3, Santiago, Chile

**Keywords:** Liquid crystals, Nonlinear phenomena, Phase transitions and critical phenomena

## Abstract

Coexistence of states is an indispensable feature in the observation of domain walls, interfaces, shock waves or fronts in macroscopic systems. The propagation of these nonlinear waves depends on the relative stability of the connected equilibria. In particular, one expects a stable equilibrium to invade an unstable one, such as occur in combustion, in the spread of permanent contagious diseases, or in the freezing of supercooled water. Here, we show that an unstable state generically can invade a locally stable one in the context of the pattern forming systems. The origin of this phenomenon is related to the lower energy unstable state invading the locally stable but higher energy state. Based on a one-dimensional model we reveal the necessary features to observe this phenomenon. This scenario is fulfilled in the case of a first order spatial instability. A photo-isomerization experiment of a dye-dopant nematic liquid crystal, allow us to observe the front propagation from an unstable state.

## Introduction

Physical systems far from the thermodynamic equilibrium are characterised by exhibiting coexistence of states^1^, that is, for the same parameters different equilibria are observed. As a consequence of the initial conditions, modification of the physical parameters, or inherent fluctuations, these systems exhibit domains between the different states. The region that separates these domains is usually called interface, wall or front, depending on the physical context under study^2^. These interfaces, in general, are propagative and can exhibit a complex dynamics. The fronts propagation phenomenon is transversal, ranging from biology, chemistry to physics. These solutions correspond to nonlinear waves. Indeed, there is no superposition principle and the interfaces have a well-defined shape. The study and characterisation of fronts have been at the core of Nonlinear Physics^1,2^. The studies of flames propagation of Faraday^3^ and gene propagation of Fisher^4^ and Kolmogorov, Petrovsky and Piskunov^5^ are pioneering works in the understanding of this phenomenon. The fronts speed depends on the stability of the connected states. In the case of connecting two stable states–bistable fronts–the more stable state invades the lesser one, with a speed proportional to the difference of energy between states^6^. Hence, by modifying a parameter one can equalize the energy between the states and the fronts become motionless, which correspond to the Maxwell point^7^. The previous scenario changes drastically when one considers fronts between a stable and an unstable state. Fronts of this type are those of the combustion process, population propagation, or permanent contagious diseases such as AIDS^8,9^. One of the main characteristics of these nonlinear waves is that the stable state invades the unstable one, that is, the combustion advances towards the non-inflamed material. Fronts between unstable states have been studied theoretically, which appear as an intermediate front between stable and unstable state, *dual fronts*^10–13^.

The purpose of this letter is to show that an unstable state generically can invade a stable one in the context of pattern forming systems. Theoretically, by means of a one-dimensional reaction-diffusion model, we have identified the minimum conditions to observe the propagation of an unstable homogenous state into a stable one. These conditions correspond to have coexistence between states, but the unstable state must be energetically more favourable than the stable one. In two spatial dimensions, this condition is satisfied in the case of a subcritical spatial instability. A prototype model of pattern formation exhibits fronts propagation from an unstable state. Based on a photo-isomerization experiment of a dye-dopant nematic liquid crystal, we can observe this front propagation.

## Results

### One-dimensional front propagation from unstable state

**Figure 1:**
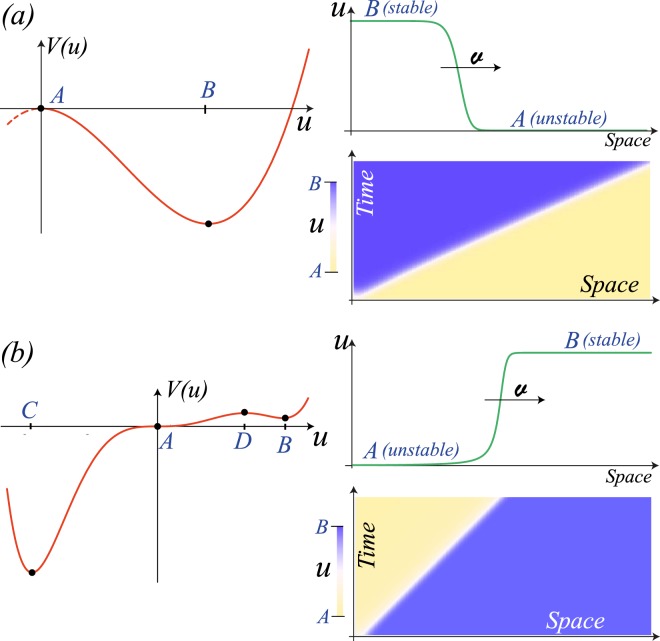
Front propagation between a stable and an unstable state. (**a**) Front propagation into unstable state, *V*(*u*) = −*u*^2^/2 + *u*^3^/3 and *ζ* = 0. *A* = 0 and *B* = 1 are unstable and stable state, respectively. The speed of propagation *v* = 2. (**b**) Front propagation from unstable state, *V*(*u*) = *u*^6^/6 − 0.7*u*^5^/5 − *u*^4^/4 + 0.7*u*^3^/3 and *ζ* = 0. *A* = 0, *B* = 1, *C* = −1, and *D* = 0.7, where *B* and *C* are stable state, *D* is an unstable state and *A* is a half stable equilibrium [15]. Left panels account for the respective potential. Right panels stand for the spatiotemporal evolution and profile of the fronts.

Let us consider an one-dimensional scalar field *u*(*x*, *t*), which satisfies a dimensionless reaction-diffusion equation

1 $${\partial }_{t}u=-\frac{\partial V}{\partial u}+{\partial }_{xx}u+\sqrt{\zeta }\xi (x,t),$$

where *V*(*u*) is a potential that characterises the dynamical evolution of *u*. Considering a potential that has coexistence between a stable and an unstable state. Figure 1a illustrates the typical potential. $\xi (x,t)$ is a gaussian white noise with zero mean value and delta correlated^14^. The parameter *ζ* accounts for the noise level intensity.

The unstable and stable equilibria are represented by symbols *A* and *B*, respectively. Stable and unstable equilibria are characterised by being a local minimum and maximum/saddle of the potential. Hence, the stable state always has less energy than the unstable equilibrium. The noiseless model Eq. (1), has a front solution that connects the equilibrium states that propagates at a constant speed in order to minimize the energy (cf. Fig. 1a)^8,9^. Let us consider a multi-stable system, which has two stable states, an unstable and a half stable equilibrium. A half stable equilibrium is a state in which one side is attractive, while on the other side is repulsive^15^. Namely, the half stable equilibrium corresponds to a nonlinear unstable saddle-point. Note that this equilibrium is non-generic because requires imposing a saddle fixed point. Figure 1b depicts an associated potential. Stable equilibria are represented by symbols *C* and *B*, the unstable and a half stable state by the symbols *D* and *A*, respectively. Depending on the initial condition, this system can present different nonlinear waves between equilibrium states. In this scenario is observed an intriguing and unexpected front that connects the stable state *B* and the saddle equilibrium *A*. Counterintuitively, the unstable stable state *A* invades the stable equilibrium *B*. Figure 1b illustrates this front propagation. The unstable state *A* invades the stable state *B* because it is more favourable energetically. Considering additive noise, we observe that the front between *A* and *B* state propagates, however at a later time an extra front appears between the stable state *C* and saddle state *A*. Finally, the state *C* invades state *A* and then state *B*. Indeed, the propagation of a front from an unstable to a stable state is a transient phenomenon because the physical system must tend to its global equilibrium. Note that, if we change the half stable state *A* for a locally stable one with the same energy, the front between *B* and *A* is unchanged. However, this scenario changes drastically if noise is considered.

**Figure 2:**
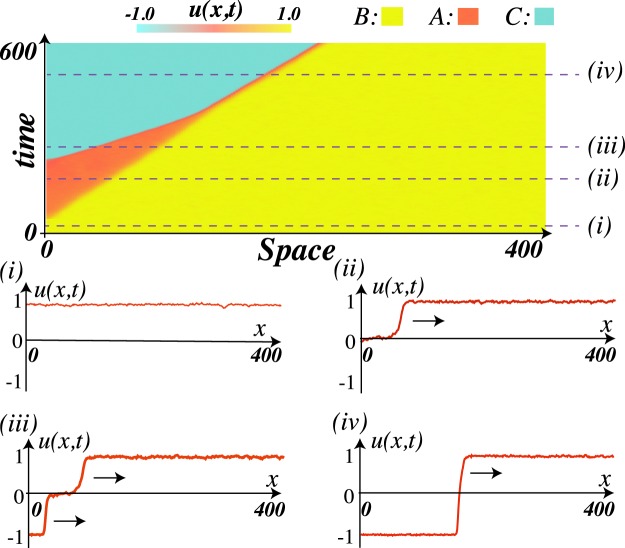
Front propagation of model Eq. (1), *V*(*u*) = *u*^6^/6 − 0.7*u*^5^/5 − *u*^4^/4 + 0.7*u*^3^/3, with additive Gaussian white noise and noise level intensity 0.1. *D* = 0.85, *B* = 1, *C* = −1, and *A* = 0 are the stable and saddle equilibrium. The upper panel shows the spatiotemporal diagram from the initial condition homogeneous solution u = 1. The lower panels account for the profile of the field *u*(*x*, *t*) at the instant represented by the (*i*), (*ii*), (*iii*), and (*iv*).

The inclusion of inherent fluctuations (*ζ* ≠ 0) in differential equations offers a more realistic description of macroscopic systems. The fluctuations are responsible for causing the blow-up of unstable equilibria, giving rise to fronts propagation. Indeed, the fluctuations generate the emergence of fronts in different spatial places^16^. The typical time of the emergence of fronts is proportional to the logarithmic of the noise level^16^. Hence, the front will be observed without interference from the fluctuations while the observation time is lower than this characteristic time. Figure 2 shows the front propagation into an unstable state obtained from the numerical simulation of model Eq. (1) with *V*(*u*) = *u*^6^/6 − 0.7*u*^5^/5 − *u*^4^/4 + 0.7*u*^3^/3 and additive Gaussian white noise. Initially, the system is prepared in the stable state *u* = 1; then a perturbation is introduced at one end of the spatial domain that induces a front between the unstable (*A*) and the stable (*B*) state. Subsequently, after the characteristic time of the fluctuations in state A, the fluctuations induce a front between the states A and C, which coexists with the front between the stable and unstable state. Later a front between the state B and C is generated (cf. Fig. 2). Multistable systems are characterised by a rich variety of fronts and dynamics among them^7,17,18^.

To observe these intriguing fronts, the system under study needs a half stable equilibrium. Hence, the system requires that at least one parameter must be set to given value. Namely, this makes the observation of these fronts between homogeneous states not so generic. As we shall show in the case of pattern formation, these fronts are generic.

### Two-dimensional front propagation from unstable state

Non-equilibrium processes often lead in nature to the formation of spatial structures developed from a homogeneous state through a spontaneous breaking of symmetries present in the system^1,2,19^. The observed patterns correspond to spatial modes that become linearly unstable, which are stabilized by the nonlinear effects. The observed wavelength can be determined by the system physical dimensions or geometrical constraints^19^. However, this wavelength can be also intrinsic, which is determined by the competition of different dynamic transport mechanisms. The origin of these patterns is often called Turing instability^20^. Several physical systems that undergo a symmetry-breaking instability close to a second-order critical point can be described by real order parameter equations in the form of Swift-Hohenberg type of models. These models, have been derived in various fields of nonlinear science such as hydrodynamics^21^, chemistry^22^, plant ecology^23^, nonlinear optics^24,25^, and elastic materials^26^. Hence, this model is the paradigmatic equation that describes patterns formation. Let us consider a generalised Swift-Hohenberg model for the real scalar field *u* = *u*(*x*, *y*, *t*), which reads^24^

2 $${\partial }_{t}u=\eta +\mu u-{u}^{3}+\nu {\nabla }^{2}u-{\nabla }^{4}u.$$

Depending on the context in which this equation has been derived, the physical meaning of the field variable could be the electric field, deviation of molecular orientations, phytomass density, or chemical concentration. The control parameter *μ* measures the input field amplitude, the aridity parameter, or chemical concentration. The parameter *η* accounts for the asymmetry between the homogeneous states. The parameter *ν* stands for the diffusion coefficient; when this parameter is negative, it induces an anti-diffusion process. This process is responsible for the emergence of patterns.

**Figure 3:**
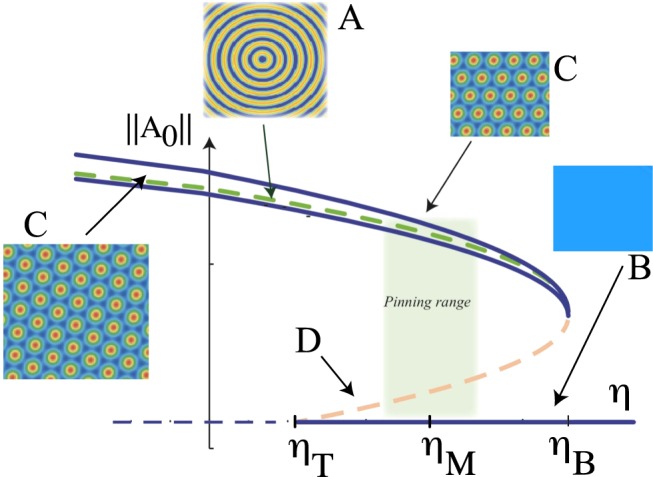
Schematic representation of the bifurcation diagram of the generalised Swift-Hohenberg model, Eq. (2) as function of *η* parameter for *ν* < 0 and *μ* < 0. The vertical axis accounts for the amplitude ||*A*_0_|| of the spatial oscillation of the pattern. *η*_*T*_, *η*_*B*_, and *η*_*M*_ account for the critical value or the transition, the nascent of bistability, and the Maxwell point, respectively. The shaded area accounts for the pinning region. Insets stand for the different equilibria, where *B*, *A*, *C*, and *D* account for the uniform stable, saddle type, hexagonal pattern, and unstable state.

For *ν* < 0 and *μ* < 0, the system only exhibits a single homogenous state. When |*η*| is large, the system is monostable. By decreasing *η* < *η*_*T*_, the system exhibits a first order spatial instability giving rise the appearance of hexagonal patterns. Hence, there is a coexistence region between the pattern and homogeneous states (*η*_*T*_ < *η* < *η*_*B*_). Figure 3 depicts the bifurcation diagram of Eq. (2) as function of the parameter *η* (for details of the bifurcation diagram see refs^22,24,27^). The vertical axis accounts for the amplitude ||*A*|| of the pattern. When the hexagons appear they can be oriented in different directions as result of the isotropy of the system (cf. Fig. 3). Another obvious spatial solution of the system corresponds to the superposition of concentric rings (see Fig. 3). However, this solution is unstable and is a saddle-type solution^28^, because the interaction of spatial modes give rise to the hexagonal patterns^19^. Likewise, numerically it has been demonstrated that the concentric rings pattern is unstable^29^. Note that localised concentric rings solutions with a small number of rings have been studied in refs^30,31^. A saddle equilibrium is characterised by being linearly marginal, nonlinear unstable, and having at least an unstable direction. Based on the mode dynamics, the states formed by many equivalent modes, which is the case of concentric rings, are generally saddle-type^19^.

**Figure 4:**
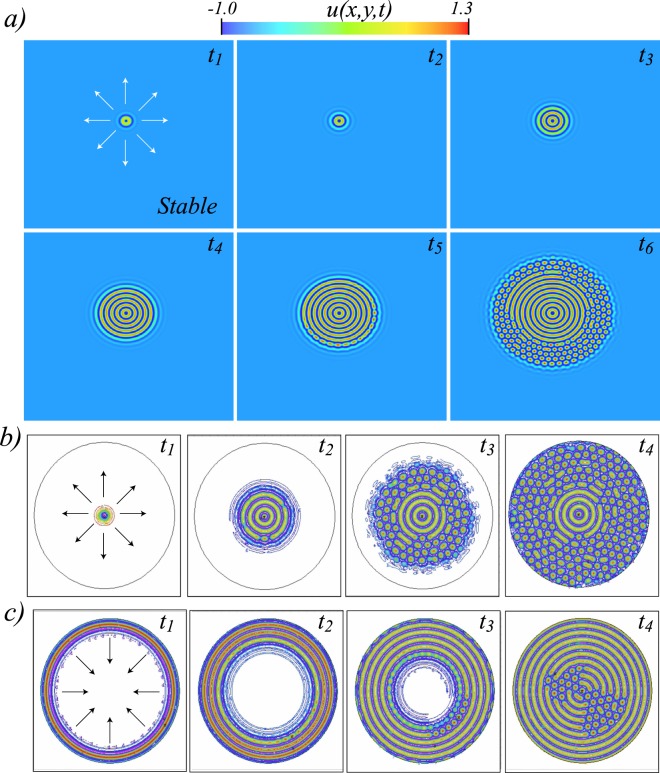
Unstable concentric ring pattern invades stable homogeneous state. Temporal sequence of numerical simulation of model Eq. (2) with *η* = −0.216, *μ* = 0.025, *ν* = −2.0, periodic (**a**) and Neumann boundary conditions (**b**,**c**) [*t*_1_ < *t*_2_ < *t*_3_ < *t*_4_ < *t*_5_ < *t*_6_].

In the coexistence region, one envisages to observe fronts between the states. Depending on the value of *η*, one state is more favourable than the other one. Both states are energetically equivalent at Maxwell point (*η*_*M*_). However, the system has a region of the parameter space where the front between these states is motionless, the *pinning range*^6^, although one state is more stable than the other one. The shaded region in Fig. 3 illustrates the pinning region. Outside this region, the most favourable equilibrium spreads on the other one. When *η* approaches *η*_*T*_ (*η* > *η*_*T*_), the homogeneous state is stable but close to becoming unstable. Then in this region of the parameter space, the unstable concentric ring pattern fulfills all the conditions to invade the stable homogeneous state. Namely, the concentric rings patterns and the homogeneous state schematically correspond, respectively, to the equilibria *A* and *B* of the potential of Fig. 1b. Figure 4 illustrates the spread of the unstable concentric rings pattern over the stable homogeneous state. Figure 4a shows this propagation considering periodic boundary conditions and as an initial condition a spot disturbance with small stochastic perturbations. Note that the spot disturbance must exceed a critical size because if it is too small, the system relaxes the uniform state. Due to the initial perturbations and the boundary conditions, the front is destabilised from a given temporal moment (*t*_4_ < *t* < *t*_5_). Generating the emergence of hexagonal patterns that propagate over the unstable state. Finally, the hexagonal pattern invades the homogeneous state^32^. However, the concentric ring pattern is pinned by the defects that are induced between both patterns (see the textbook^2^ and reference therein). Note that pinning defects are responsible for generating the richness of textures observed in spatial patterns. A similar phenomenon is observed, when one considers a circular domain disturbed at the centre with Neumann boundary condition (cf. Fig. 4b). Likewise, we have considered a ring-shaped disturbance at the edge of the domain to analyse how the front penetrates into the inside of the domain (see Fig. 4c). Therefore, a stable extended state is invaded by an unstable pattern. Note that the difference between the dynamics observed in one and two spatial dimensions are that the secondary front that connects the two stable states invades the entire system (one-dimension) and partially (two-dimensions) due to the existence of defects.

### Experimental front propagation from unstable state in photo-isomerization process in a dye-doped nematic liquid crystal layer

**Figure 5:**
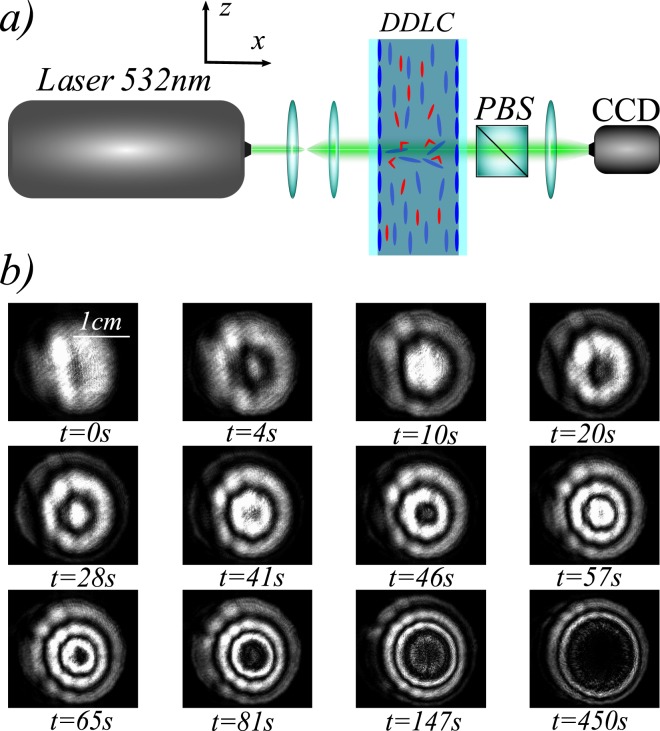
Concentric ring pattern propagation in photo-isomerization process in a dye-doped nematic liquid crystal layer illuminated by a laser beam with a Gaussian profile. (**a**) Schematic representation of experimental setup, DDLC: dye-doped nematic liquid crystal cell, PBS: polariser beam-splitter, and CCD: charge-coupled camera device. (**b**) Temporal sequence of concentric ring pattern propagation.

To experimentally observe front propagation from an unstable state, we consider the photo-isomerization process in a dye-doped nematic liquid crystal layer illuminated by a laser beam with a Gaussian profile. For high enough input power, a phase transition from the nematic to the isotropic state takes place in the illuminated area and then the two phases are spatially connected via a front propagating outward from the centre of the beam^33^. For lower input power, photo-isomerization can induce patterns, which correspond to the spatial modulation of the molecular order. Figure 5 depicts the experimental setup under study. Theoretically was demostrated, recently, that an equivalent model to Eq. (2) describes the photo-isomerization process in a dye-doped nematic liquid crystal layer, where *u* stands for the Landau-De Gennes molecular scalar order parameter (see the details in^34^ and the equivalence of the models in Supplementary Material).

The cell consists of two glass plates coated with Poly-Vinyl-Alcohol and rubbed to favour the planar alignment of the liquid crystal molecules, with a separation of 25 *μm*. The gap is filled with an E7 nematic liquid crystal doped with the azo-dye Methyl-Red at a concentration of 0.75% by weight. To induce the fronts, the cell is irradiated with a frequency doubled Nd^+3^:YVO_4_ laser, polarised in the vertical direction with wavelength *λ*_0_ = 532 *nm* in the absorption band of the dopants. A polariser beam-splitter is placed in between the liquid crystal sample and the CCD camera to distinguish the molecular orientation in the sample. Two planoconvex lenses increase the laser beam diameter to 2 *cm*. The cell was subjected to input powers between *P* = 300 and *P* = 700 mW.

Applying a light beam on the sample creates the gradual emergence of concentric rings that propagate from the centre of the beam to outside. Figure 5b displays a temporal sequence of the unstable concentric rings propagation. Near the boundary of the illuminated region, the rings begin to deform with a similar morphology that observed in the numerical simulations. However, hexagonal patterns are not observed, since the phase that finally invades the system is the isotropic liquid state that corresponds to the black zone within the illuminated domain. This region is black since light can not cross an isotropic medium between crossed polarisers. Since this stable state is homogeneous, no trace of the concentric rings remains. In the event that the final state is a pattern, there will always be a trace of the front between the stable and the unstable state (cf. Fig. 4). Some rings are observed in experimental^35^ or in nature^36^, which is the *footprint* that there was an unstable state that invaded a stable one.

## Discussion

Pattern formation has been observed in diverse contexts that are ranging from chemistry, biology, and physics. Hence, in subcritical spatial instabilities, one expects to find transients of ring-shaped patterns that propagate into a stable uniform state. Namely, an unstable spatial state can invade a stable one. This phenomenon is a consequence of the concentric rings being an unstable saddle state that may have lower energy than a uniform stable state. These are the necessary elements to observe this counterintuitive phenomenon. Front propagation is a transient phenomenon with the aim of establishing the prevalence of an equilibrium in the system under study. If the front connects an unstable state, it makes the front fragile in the face of imperfections, boundary conditions, and fluctuations. Hence, it is complicated to observe experimentally. However, we show that the propagation of unstable concentric rings over a stable uniform state, as a result of the dynamics of defects, always leaves an imprint of rings (bullseye patterns), which can be observed and make the phenomenon relevant to explain the diversity of textures in out of equilibrium systems.

## Methods

### Numerical methods

One dimensional numerical simulations were performed using Runge-Kutta order 4 with 400 points in space and Neumann boundary conditions. *dx* = 0.5 and *dt* = 0.1 were used in the discretisation scheme.

Two dimensional model numerical simulations were performed using Runge-Kutta order 4 with a rectangular grid with 512 × 512 with *dx* = *dy* = 0.5 and *dt* = 0.06 points and with triangular finite elements in order to create a quasi circular boundary condition or radius 90 points in the grid and Neumann border conditions.

Values of the parameters used to perform the numerical integration were given in the corresponding caption.

### Experimental methods

Nematic liquid crystals are characterised by having a rod-like anisotropic molecular structure, that is, these molecules are distinguished by having a uniaxial structure. In certain temperature range, these molecules are locally aligned forming the nematic phase (thermotropic liquid crystal)^37–39^. To substantially increase the coupling between the light and the nematic liquid crystal dye-dopants are added to a liquid crystal matrix host. The dye-dopant molecules should have a uniaxial rod-like structure^38^, which is not necessarily a liquid crystal. Likewise, the concentration in weight of the dye-dopant in the liquid crystal must be low in order to not degrade the properties of the liquid crystal and ensure the solubility of the mixture. In the case of E7 liquid crystal and methyl-red dye, the experiments were performed in mixtures in the range of 0.25% up to 1% concentration by weight. Here we reported the case of 0.75% concentration by weight.

The experimental setup is depicted in Fig. 5(a). A dye-doped nematic liquid crystal (DDLC) cell subjected to an orthogonal Gaussian laser beam is studied. The cell was filled with an E7 nematic liquid crystal doped with the azo-dye Methyl-Red at a concentration of 0.75% in weight. The elastic constants of the liquid crystal under consideration are, respectively, *K*_1_ = 11.2, *K*_2_ = 6.8, and *K*_3_ = 18.6 (×10^−12^*N*) and the relative parallel and perpendicular dielectric constants are ${\varepsilon }_{\parallel }=18.96$ and *ε*_⊥_ = 5.16. The cell consists of two glass plates coated with Poly-Vinyl-Alcohol (PVA) and rubbed to favour the planar alignment of the liquid crystal molecules, nematic director parallel to the substrates. The cell is a sandwich type with *d* = 25 *μm* thick spacers. The gap is filled with the dye-doped nematics liquid crystal. This type of configuration favours the dopant molecules to be positioned with different orientations, which ensures a relevant coupling with the light that crosses the sample. Figure 5(a) illustrates schematically the molecules when the sample is not illuminated. To induce the rings, the cell is irradiated with a frequency doubled Nd^+3^:YVO4 laser, with wavelength *λ*_0_ = 532 *nm* in the absorption band of the dopants, and with vertical polarization (following y-axis, cf. Fig. 5(a). The cell was subjected to input powers between *P* = 300 *mW* and *P* = 700 *mW*. Two bi-convex lenses increase the laser beam diameter to 2 *cm*. Additionally, a linear polarized beam spliter (PBS) is positioned at the output of the dye-doped nematic liquid crystal sample to analyse the response of the light that crosses the cell. Likewise, the polariser PBS (analyser) can be rotated with respect to the laser polarisation direction to characterise the birefringence properties of the liquid crystal sample. The transmitted beam is recorded with a CCD camera (Thorlabs DCU224M, 1280 × 1024 pixels). An ocular lens is placed between the PBS and the CCD camera to achieve a better imaging.

## Supplementary information

Supplementary information 
