# Supplementary material for: Extended stable equilibrium invaded by an unstable state
Source: Sci Rep. 2019 Oct 22;9:15096. doi: 10.1038/s41598-019-51064-5 (PMC6805852; doi:10.1038/s41598-019-51064-5)
Supplement: Supplementary file 1 — Supplementary information [file 41598_2019_51064_MOESM1_ESM.pdf]

Extended stable equilibrium invaded by an unstable state

Stability of concentric rings pattern in the generalised Swift-Hohenberg equation

Camila Castillo-Pinto, Marcel G. Clerc, Gregorio González-Cortés

Physics Department and Millennium Institute for Research in Optics,
Facultad de Ciencias Físicas y Matemáticas,
Universidad de Chile, Casilla 487-3, Santiago, Chile.

September 19, 2019

1 Photoisomerization of dye-doped liquid crystal cell: a simplified model

Let us consider a dye-doped liquid crystal cell modeled by [1]

$$\begin{aligned}\partial_t S =& -\left(A+\beta C_0\left(I\right)\right)S+\left(B+\alpha\beta\right)S^2-S^3+\frac{D^2}{\lambda}\nabla^4 S\\ &+\left(1-D\alpha\right)\nabla^2-\frac{\alpha}{\lambda}\left(\delta_\parallel\partial_{xx}+\delta_\perp\partial_{yy}\right)\nabla^2 S, \qquad (1)\end{aligned}$$

where S is the scalar order parameter that describes the degree of alignment of molecules. A and B are phenomenological parameters that characterise the nematic-isotropic transition in the Landau-De Gennes theory. D stands for the elastic coupling. $\delta_\parallel$ and $\delta_\perp$ are diffusion coefficients of the concentration of the *cis-state* dopant in the parallel and orthogonal direction with respect to the incident polarization of the electric field, respectively. λ is the decay rate related to the transition from *cis* to *trans* state by thermal relaxation. C_0 is the equilibrium concentration of dye molecules in the *cis-state*, which is a function of the incident light intensity I. α accounts for the reduction of the *cis-state* concentration when liquid crystal is more aligned (large S). β stands for the entropic effect of the photoisomerization process. In the case of a planar alignment, the constants $\delta_\perp$ and $\delta_\parallel$ are similar[2]. Hence, one can approach $\delta_\perp=\delta_\parallel=\delta$. Equation (1) can be rewritten as (the generalised Swift-Hohenberg equation)

$$\partial_t v=\epsilon v-\kappa v^2-v^3-\nu\nabla^2 v-\nabla^4 v, \qquad (2)$$

with the field $v = S$, and scaling the space by a factor $\lambda\,/\,(D - \alpha\delta)$, and defining the parameters $\epsilon \equiv -\left(A + \beta C_0(I)\right)$, $\kappa \equiv (B + \alpha\beta)$, and $\nu \equiv (1 - D\alpha)\,\sqrt{\lambda/\left(D^2 - \alpha\delta\right)}$.

Therefore, a dye-doped liquid crystal cell with planar anchoring is described by model equation (2).

2 Equivalence between two forms of the generalised Swift Hohenberg equation

To study the stability of concentric ring patterns, we will analyse the dynamic close to spatial instability through the use of amplitude equations. Let us consider the generalized Swift-Hohenberg equation

$$\partial_t u = \eta + \mu u - u^3 - \nu \vec{\nabla}^2 u - \vec{\nabla}^4 u. \tag{3}$$

Considering the change of variable $v(\vec{r}, t) = u(\vec{r}, t) + u_0$, where u_0 satisfies the equation $\eta + \mu u_0 + u_0^3 = 0$, equation (3) can be rewritten as

$$\partial_t v = \varepsilon v - \kappa v^2 - v^3 - \nu \vec{\nabla}^2 v - \vec{\nabla}^4 v, \tag{4}$$

with $\kappa \equiv 3u_0$ and $\varepsilon \equiv \mu + u_0^2$. It is easy to see that $v = 0$ is a solution. Since both models are equivalent, we will use model (4) to analyze the stability of concentric rings.

3 Stability of concentric ring patterns on imperfect Swift Hohenberg Equation

To study the stability of this solution, we consider the ansatz $v(\vec{r}, t) = \bar{v} e^{\sigma t - i\vec{k}\cdot\vec{r}}$ in eq. (4. After straightforward calculation one obtains the growth rate relation

$$\sigma = \varepsilon + \nu \vec{k}^2 - \vec{k}^4. \tag{5}$$

Note that if $\sigma(\vec{k})$ is positive (negative) the zero solution is linearly unstable (stable). Hence, the system exhibits a spatial bifurcation for $\varepsilon = -\nu/4$ characterized by a critical wavenumber $k_c = \sqrt{\nu/2}$. Close to the bifurcation the equation reads

$$\partial_t v = \left(-\frac{\nu}{4} + \Delta\epsilon\right) v - \kappa v^2 - v^3 - \vec{\nabla}^2 v - \vec{\nabla}^4 v, \tag{6}$$

where $\Delta\epsilon \equiv \varepsilon + \nu/4$ is the spatial bifurcation parameter. When increasing the bifurcation parameter, the system exhibits the formation of hexagonal and concentric ring patterns. Due to the rotation symmetry of the concentric ring patterns, the previous equation can be written in polar coordinates

$$\begin{aligned}\partial_t v = &\left(-\frac{\nu}{4} + \Delta\epsilon\right) v - \kappa v^2 - v^3 - \nu\left(\partial_{rr} + \frac{\partial_r}{r} + \frac{\partial_{\theta\theta}}{r^2}\right) v \\ &- \left(\partial_{rrrr} + \frac{2\partial_{rrr}}{r} + \frac{2\partial_{rr\theta\theta}}{r^2} - \frac{\partial_r r}{r^2} - \frac{2\partial_{r\theta\theta}}{r^3} + \frac{\partial_r}{r^3} + \frac{4\partial_{\theta\theta}}{r^4} + \frac{\partial_{\theta\theta\theta\theta}}{r^4}\right) v. \end{aligned} \tag{7}$$

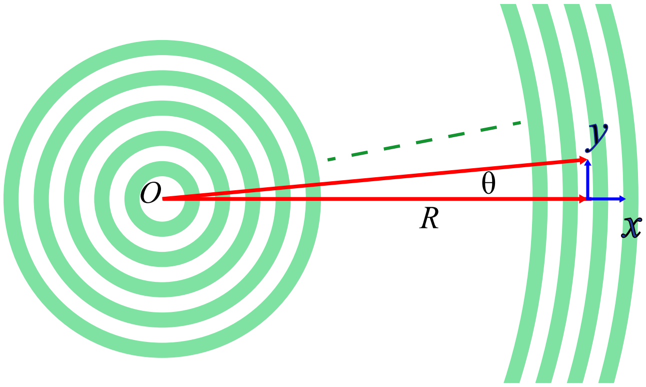

Figure 1: Schematic representation of concentric rings patterns and the asymptotic aligned reference coordinate system $\{x, y\}$. Point O accounts for the origin of polar coordinates and center of concentric rings. R accounts for a radial vector sufficiently far from the origin and with a magnitude much larger than the size the typical length of the patterns ($R\sqrt{\nu} \gg 1$).

Since an expression for concentric ring patterns is not analytically known, one strategy to study its stability is to asymptotically analyze its stability away from the origin of the rings ($r \gg 1$). Hence, we introduce the asymptotic aligned coordinate system with a ring [3], as it is depicted in Fig. 1. In this local coordinates systems, the concentric ring pattern is periodic along the x coordinate, and its variations in y coordinates are small. Polar and asymptotic local asymptotic coordinates are related by $r = R + x$ and $y = R\theta$. Replacing this asymptotic local coordinates in Eq. (7), we obtain a dominant order

$$\partial_t v(x,y,t) = -\frac{\nu}{4}v + \Delta\epsilon v - \kappa v^2 - v^3 - \nu\partial_{xx}v - \frac{\nu}{R}\partial_x v - \nu\partial_{yy}v$$
$$-\left(\partial_{xxxx} + \frac{2\partial_{xxx}}{R} + 2\partial_{xxyy} - \frac{2\partial_{xyy}}{R} + \partial_{yyyy}\right)v + O\left(\frac{1}{R^2}\right). \quad (8)$$

Based on weakly nonlinear analysis, one can introduce the ansatz [4]

$$v(x,y,t) \approx A(X,Y,T)e^{i\sqrt{\frac{\nu}{2}}x} - \frac{8\kappa}{\nu^2}|A|^2 - \frac{4\kappa}{9\nu^2}A^2 e^{i2\sqrt{\frac{\nu}{2}}x} + c.c., \quad (9)$$

where A is the slow variable amplitude of the pattern, X, Y, and T account for the slow spatial and temporal scales, into Eq. (8), and considering the dominant resonant terms, one gets

$$\partial_T A = \Delta\epsilon A - \left(3 - \frac{152\kappa^2}{9\nu^2}\right)A|A| + 2\nu\left(\partial_X - \frac{i}{\sqrt{2\nu}}\partial_{YY}\right)^2 A. \quad (10)$$

Defining the parameter $q \equiv \sqrt{\nu/2}$, rescaling $x = \sqrt{\nu}x'$, $y = \nu^{1/4}y'$, and the amplitude $C \equiv \sqrt{\left(3 - \frac{152\kappa^2}{9\nu^2}\right)}A$, then we obtain the Newell-Whitehead-Segel

equation [5, 6]

$$\partial_t C = \Delta\epsilon C - C\,|C| + \left(\partial_x - \frac{i}{2q}\partial_{yy}\right)^2 C. \tag{11}$$

This amplitude equation has a family of stationary stripe solutions of the form $C = \sqrt{\Delta\epsilon - p^2}e^{ipx}$. Close to the spatial bifurcation of Eq. (3), the concentric rings pattern are asymptotically described by the homogeneous solution $p = 0$. To analyze the stability of these solutions, we consider the ansatz

$$C = \left(\sqrt{\Delta\epsilon - p^2} + a_+ e^{i\vec{Q}\vec{r}} + a_- e^{-i\vec{Q}\vec{r}}\right) e^{ipx}, \tag{12}$$

where $\vec{Q}$ is an arbitrary wavevector with small magnitude. Replacing the above ansatz in Eq. (11) after straightforward calculations, one gets

$$\partial_t a_+ = -\left(\epsilon - p^2 - \left((p+Q_x)^2 - \frac{Q_y^2}{q}p - p^2\right)\right) a_+ - \left(\epsilon - p^2\right) a_-, \tag{13}$$

$$\partial_t a_- = -\left(\epsilon - p^2 - \left((p-Q_x)^2 - \frac{Q_y^2}{q}p - p^2\right)\right) a_- - \left(\epsilon - p^2\right) a_+. \tag{14}$$

Defining $\gamma^2 \equiv \epsilon - p^2$ and $U_\pm \equiv \left((p \pm Q_x)^2 - p^2 - \frac{Q_y^2}{q}p\right)$, this set of equations reads

$$\partial_t a_+ = -\left(\gamma^2 - U_+\right) a_+ - \gamma^2 a_-, \tag{15}$$

$$\partial_t a_- = -\left(\gamma^2 - U_-\right) a_- - \gamma^2 a_+. \tag{16}$$

Considering perturbation in the orthogonal direction $\vec{Q} = Q_y\hat{y}$ ($U \equiv U_+ = U_- = Q_y^2 p/q$) and introducing solutions of the form $a_\pm = \delta e^{\sigma t}$, the growth rate satisfies $(\gamma^2 + U) + \sigma = \pm\gamma^2$. Namely, one has two solutions $\sigma_+ = pQ_y^2/q$, $\sigma_- = pQ_y^2/q - 2\gamma^2$. Therefore, the primary mode $p = 0$ has a marginal direction in the functional space with a null eigenvalue. Indeed, the ring solution is marginally stable. Likewise, numerical analysis shows that concentric rings patterns are linearly unstable [7].

References

[1] Andrade-Silva I. , Bortolozzo, U., Clerc, M. G., González-Cortés, G., Residori, S., and Wilson, M. . *Spontaneous light-induced Turing patterns in a dye-doped twisted nematic layer*. Scientific reports, **8** (2018).

[2] Andrade-Silva I., Bortolozzo U., Castillo-Pinto C., Clerc M. G., González-Cortés G., Residori S. and Wilson M. *Dissipative structures induced by photoisomerization in a dye-doped nematic liquid crystal layer*. Phil. Trans. R. Soc. A **376**:20170382 (2018)

[3] L.M. Pismen, *Patterns and interfaces in dissipative dynamics* (Springer Science & Business Media, 2006).

[4] P. Manneville, *Dissipative Structures and Weak Turbulence* (Academic, New York, 1990).

[5] A.C. Newell and J. A. Whitehead. J. Fluid Mech. **38**, 279403 (1969).

[6] A.L. Segel. J. Fluid Mech. **38**, 203 (1969).

[7] D. Lloyd, and B. Sandstede, Nonlinearity, **22**, 485 (2009).
